# Study on the Effect of Key Genes *ME2* and *adhE* during *Luzhou-Flavor Baijiu* Brewing

**DOI:** 10.3390/foods11050700

**Published:** 2022-02-26

**Authors:** Wen Zhou, Yu Xia, Yajiao Zhao, Yan Wang, Zhengyun Wu, Taikei Suyama, Wenxue Zhang

**Affiliations:** 1 College of Biomass Science and Engineering, Sichuan University, Chengdu 610065, China; zhouw103077@126.com (W.Z.); xiayu_scfs@163.com (Y.X.); zhaoyj0730@163.com (Y.Z.); summer_wy94@163.com (Y.W.); wuzhengyun@scu.edu.cn (Z.W.); 2 Department of Light Industry Engineering, Sichuan Technology and Business College, Dujiangyan 611800, China; 3 National Institute of Technology, Akashi College, Akashi 674-8501, Japan; suyama@akashi.ac.jp; 4 School of Liquor-Making Engineering, Sichuan University Jinjiang College, Meishan 620860, China

**Keywords:** *Luzhou-flavor baijiu*, gene *ME2*, gene *adhE*, functional microorganisms, flavors

## Abstract

*Luzhou-flavor baijiu* (LFB) is brewed by the combined action of various microorganisms, and its flavor is affected by the microbial community and the genes they express, but which genes are the key ones during LFB brewing is less clear. Based on our previous studies the genes *ME2* and *adhE* were identified as key genes, but which role they play was also unknown. In this study functional microorganisms were screened based on the key genes *ME2* and *adhE*, and they were identified to be *Rummeliibacillus suwonensis*, *Clostridium tyrobutyricum* and *Lactobacillus buchneri*. Then simulated fermentation experiments were carried out with the functional microorganisms, and during the fermentation process expression of the key genes and the amounts of the main flavors were detected to analyze the role of the key genes. The results showed that the key gene *ME2* was significantly positively correlated with the contents of the main acids, however the key gene *adhE* and the formation of the main esters in the LFB brewing process was a significant positive correlation. This study verified the two key genes *ME2* and *adhE* complement each other in the LFB brewing process, playing an important role in promoting the formation of flavor substances, and are very beneficial to improve the quality of LFB.

## 1. Introduction

*Luzhou-flavor baijiu* (LFB) is one of the most popular baijiu products in China [1], due to its rich and comforting aroma and flavor. In 2020, its production and sales volume accounted for more than 50% of the total volume of baijiu. The main factors affecting the flavor of LFB are the fermentation microorganisms in *Daqu* (fermentation starter), the pit mud (mud attached to the underground cellar surface, which was used for *Luzhou-flavor baijiu* fermentation) and *zaopei* (fermented grains mixture). Therefore, in recent years, an increasing number of studies on LFB brewing have focused on the analysis of the microbial community structure, gene expression and metabolic pathways in *zaopei*, *Daqu*, and pit mud [2,3,4,5,6].

Key genes revealed to be important in the LFB brewing process were expressed by key microorganisms. Screening and fermentation research on these key microorganisms is an important method to analyze the mechanism of key genes. For a long time, researchers have been screening beneficial microorganisms from pit mud [7], *Daqu* [8] and *zaopei* [9], and some researchers even proposed the method of culturomics [10] to study the characteristics of microorganisms in baijiu production. Most of these beneficial microbes were screened based on some specific function (such as acid producing, saccharification, cellulose degradation), which can be used to improve the LFB brewing process. This function-oriented screening method is beneficial to the application of microorganisms, but the traditional screening method has some problems such as strong randomness and high screening intensity. Recently, methods of screening microorganisms based on genetic information have been gradually applied. Some studies have determined the microbial community information of pit mud based on 16S rRNA gene information then designed corresponding media for key microorganisms screening [10]. Also, there were studies on screening new species from pit mud based on 16S rRNA gene information [11]. Even some studies according to the prediction function of 16S rRNA gene diversity analysis predicted the species origin of key genes, then GRPWREC tool of KOMODO database was used to predict the screening medium based on the 16S rRNA gene information of microorganisms, and finally the key gene expression strains were screened [12]. These screening methods based on microbial genetic information are speedy and accurate.

**Figure 1:**
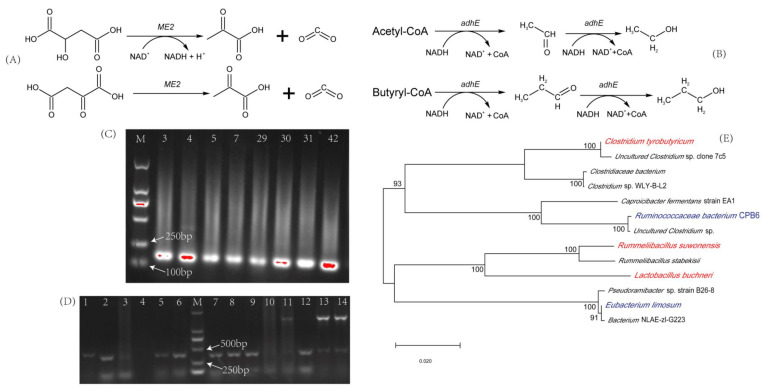
(**A**) The two biochemical reactions catalyzed by malate dehydrogenase (enzyme expressed by gene *ME2*); (**B**) The two biochemical reactions catalyzed by acetaldehyde dehydrogenase (enzyme expressed by gene *adhE*); (**C**) Agarose gel electrophoresis of the products of colony PCR based on gene *ME2*; (**D**) Agarose gel electrophoresis of the products of colony PCR based on gene *adhE*; (**E**) Phylogenetic tree of the functional microorganisms based on 16S rDNA gene sequences.

Compared with genetic information on the DNA level, mRNA information can more directly reflect the expression of functional genes. However, there were few reports on targeted screening of functional microorganisms based on mRNA information, and especially in the production of LFB this has never been reported. In previous studies, our team analyzed the differentially expressed genes between aged pit mud and degenerated pit mud used for LFB brewing by metatranscriptomics [13], and the genes *ME2* and *adhE* were found to be the most differentially expressed functional genes, which may be related to the flavor of LFB. The malate dehydrogenase expressed by gene *ME2* is listed in the Kyoto Encyclopedia of Genes and Genomes (KEGG) and mainly catalyzes two reactions (Figure 1A), the product of these two reactions are pyruvate. Pyruvate has been proved to be an important substrate for the synthesis of the “four dominant acids” (L-lactic acid, acetate, butyrate and caproate) in previous studies [13], therefore, the expression of gene *ME2* may play an important role in promoting the formation of the “four dominant acids”. *Methanogenic archaea*, *A. fulgidus* [14], *Bacillus subtilis* [15] and other common microorganisms in the LFB brewing process have also been reported to be able to express malate dehydrogenase. However, the role of the malate dehydrogenase gene (*ME2*) in the brewing process of LFB is still unknown, and as a key gene found in previous studies, its influence on the flavor of LFB needs further study. Acetaldehyde dehydrogenase gene (*adhE*) has been reported to be related to the synthesis of ethanol and butanol (Figure 1B) [16,17], and may lead to the reduction of the synthesis of acetic acid and butyric acid [18]. The reduction of gene *adhE* expression will lead to the reduction of ethanol synthesis and the increase of hydrogen production [19], due to the existence of “interspecific hydrogen transfer”, hydrogen production can promote the production of caproic acid by caproic acid bacteria [4]. However, the role of acetaldehyde dehydrogenase gene (*adhE*) in the brewing process of LFB has not been reported, especially the influence of the formation of “four dominant acids” and “four dominant esters” (ethyl lactate, ethyl acetate, ethyl butyrate and ethyl caproate), which have great influence on the flavor of LFB, is also unknown.

In this study, the species origin was aligned according to the RNA sequence information of the genes *ME2* and *adhE*, then the optimal screening medium was predicted by GRPWREC tool according to the 16S rRNA sequence information of the species, next, the functional microorganisms containing genes *ME2* and *adhE* were screened from the aged pit mud. Finally, fermentation experiments were carried out to determine the effects of functional microorganisms on the formation of main flavor substances in the LFB brewing process.

## 2. Materials and Methods

### 2.1. Sampling Procedure

Aged pit mud used for functional microorganisms screening was sampled from a LFB manufacture located in Qionglai (Sichuan, China). About 500 g of aged pit mud was collected from the bottom of the cellar, then it was sealed into a sterile bag and stored in ice bags, next, it was transported to our laboratory and stored in the refrigerator at 4 °C for further use.

### 2.2. Screening Media Prediction

The detected RNA sequences of gene *ME2* and *adhE* by metatranscriptomics were aligned to National Center for Biotechnology Information (NCBI) to identify the source of microbial species, then 16S rRNA gene sequences of the species which contain gene *ME2* and *adhE* were searched in NCBI. Next, the 16S rRNA gene sequences were imported into the GRPWREC tool of KOMODO (komodo.modelseed.org/growrec.htm, accessed on 17 September 2020) to predict the screening media of the functional microorganisms. According to the prediction results, the media with the highest score (DSMZ_330 and DSMZ_614) were selected to execute the functional microorganisms screening.

### 2.3. Enrichment and Screening of Functional Microorganisms in Pit Mud

DSMZ_330 and DSMZ_614 liquid media (the preparation method is hown in Table S1) were prepared and 15 mL were subaliquoted into anaerobic culture tubes (φ16 × 125 mm), then 1 g of aged pit mud was weighed and quickly put into the media and sealed for microorganisms enrichment. The enrichment was performed at 37 °C for 10 days. Syringes were used to pierce the sealant pad of anaerobic culture tube, from which 0.5 mL enriched cultures was extracted, then they were quickly spread at DSMZ_330 and DSMZ_614 solid medium plates. The spread plates were placed in an anaerobic incubator, and an AnaeroPack (Mitsubishi Gas Chemical Company, Tokyo, Japan) were placed in the same anaerobic incubator to maintain the internal anaerobic state, cultured at 37 °C for 7 days until colonies appeared.

### 2.4. Colony PCR

According to the sequence information of gene *ME2* and *adhE*, the software Primer Premier 5.0 (premier biosoft, San Francisco, CA, USA) was used to design specific primers for colony PCR. The designed primers (shown in Table 1) were aligned to NCBI to verify their specificity.

Colonies with bacterial characteristics grown on the plate were randomly selected with a pipette tip, then dissolved in 10 μL sterile deoxidized double distilled water, 2 μL bacterial solution was taken as a PCR amplification template. Colony PCR was carried out in a MyCycler™ thermal cycler (Bio-Rad, Hercules, CA, USA) with corresponding primers according to the procedure shown in Table 1. PCR products were detected by agarose gel electrophoresis with a concentration of 1.2%. The bands with correct electrophoresis positions were cut and purified, then sequenced by Tsingke Biotechnology Co., Ltd. (Beijing, China), and the sequencing results were aligned in NCBI. The remaining 8 μL bacterial solution was transferred to 2 mL anaerobic liquid media and cultured in an anaerobic culture bag (Mitsubishi Gas Chemical Company) at 37 °C for 5 days.

### 2.5. Functional Strains Identification and Phylogenetic Tree Construction

The colonies which were verified by colony PCR were cultured, then their 16S rRNA genes were paired-end sequenced at Tsingke Biotechnology Co., Ltd. The 16S rRNA gene sequences were aligned to NCBI through BLASTN, then the species information of the screened functional strains was determined according to homology sequences with the highest similarity. Next, the 16S rRNA gene sequence of the strains with high similarity to the screened functional strains were downloaded from NCBI, and the phylogenetic tree was constructed by neighbor joining (NJ) method with MEGA-X [20].

### 2.6. Fermentation Test of Functional Microorganisms

Solid fermentation medium: *zaopei* (a solid mixture of fermented grains include sorghum, corn, wheat and rice, which can produce LFB after distillation) was used as solid fermentation medium. The water, starch and reducing sugar contents of which was 61.2–62.5%, 10.6–11.2% and 0.48–0.52%, respectively, and its acidity was 0.38–0.40 mmol/g. The *zaopei* was taken from a LFB manufacture located in Chengdu (Sichuan, China).

Preparation of liquid fermentation medium: the *zaopei* which was just finished fermenting and dug up from the fermentation cellar was mixed with boiled distilled water at a mass ratio of 1:3. The mixture was extracted by ultrasonic in a 45 °C water bath for 120 min, then the supernatant was taken after centrifugation at 3000 rpm for 5 min. Finally, supernatant was boiled with nitrogen for 30 min to deoxidization and then stored at 4 °C for later use. Before use, it was mixed with seed media of different strains in equal volume and autoclaved at 121 °C for 20 min.

DSMZ_330 or DSMZ_614 medium was used as seed medium, and 10% of the screened functional microorganisms were inoculated in anaerobic culture tube for 5 days activation culture at 37 °C. The activated functional microorganisms were inoculated into liquid fermentation medium at a rate of 10% and fermented in an anaerobic culture flask at 37 °C for 27 days. The fermentation broth of just inoculated mixture (day 0), day 1, day 5, day 13, day 23 and day 27 was taken to detect the expression quantity of key genes and main flavor substances. The uninoculated sterile liquid fermentation medium was used as blank control.

*Zaopei* (200 g) was charged to a 500 mL conical flask and pressed tightly. Then the activated functional microorganisms were inoculated into *zaopei* at 5% and 10% inoculation rates, respectively, and the flask sealed with one-way valves for 35 days of anaerobic fermentation at 37 °C. The main flavor substances in fermented *zaopei* of day 0, day 2, day 6, day 13, day 20 and day 35 were detected. *Zaopei* which was inoculated with sterile medium were used as a control sample.

### 2.7. Quantify the Expression Quantity of Gene ME2 and adhE

Two mL of fermentation liquid was taken, centrifuged at 4 °C 10,000 rpm for 3 min, and the precipitate was immediately extracted with TR205 total RNA rapid extraction kit (Tianmo Biotech, Beijing, China) according to the method described in its instructions. The extracted RNA was detected by agarose gel electrophoresis with a concentration of 1.0% to verify their integrity, then the RNA concentration were detected by Nanodrop 2000c ultramicro spectrophotometer (Thermo Fisher Scientific, Waltham, MA, USA). All the checked RNA were used to synthesize cDNA by the kit 5X All-In-One RT MasterMix with AccuRT (abmgoodchina Inc., ZhenJiang, China).

The method of real-time fluorescence quantitative PCR (RT-qPCR) was used to detect the expression quantity of gene *ME2* and *adhE* in a LightCycler^®^ Nano System (Roche, Basel, Switzerland). RT-qPCR were carried out in a total volume of 20 µL containing BlasTaqTM 2X qPCR MasterMix (abmgoodchina Inc.) 10 µL, forward and reverse primer (Table 2) 0.5 µL, respectively, cDNA template 2 µL, and RNase-free ddH_2_O 7 µL. The RT-qPCR procedure was initial denaturation at 95 °C for 600 s; followed by 40 cycles of denaturation at 95 °C for 15 s, annealing at 52 °C for 90s.

Standard curve creation: Standard plasmids containing gene *ME2* and *adhE* were constructed. After concentration determination, the plasmids were diluted by 10-fold gradient to obtain plasmids with a concentration of 10^12^–10^7^ copies/mL. Different concentrations of plasmids were used as templates for qPCR, according to the logarithm of plasmids concentration and the Ct value of qPCR amplification, the linear fitting was carried out to draw the standard curve.

### 2.8. Detection of Volatile Flavor Substances

The volatile compounds in the fermentation samples were detected by gas chromatography-mass spectrometry (GC-MS).

Sample treatment: The liquid fermented samples were centrifuged at 4 °C 10,000 rpm for 3 min, 5 mL of supernatants were taken and put into an Agilent headspace injection bottle (Agilent Technologies, Inc., Santa Clara, CA, USA), mixed with 3.0 g NaCl and 200 μL n-octanol standard (0.55mg/L) and sealed. The solid fermentation samples were weighed 5.00 g and put into the Agilent headspace injection bottle, 8 mL ddH_2_O, 3.0 g NaCl and 200 μL *n*-octanol standard (0.55 mg/L) were added into the bottle, then sealed the bottle. After sealing, the sample bottle was heated for balanced at 95 °C for 5 min in Agilent 7697A headspace sampler (Agilent Technologies, Inc., Santa Clara, CA, USA), then the balanced gas was extracted for injection.

Chromatography and mass spectrometry conditions: A GCMS-QP2010 SE system (Shimadzu Co., Ltd., Kyoto, Japan) was used for GC-MS detection. The chromatographic column was KB-5MS capillary column (30 m × 0.25 mm, 0.25 μm); Injector temperature: 250 °C; Carrier gas: helium (purity 99.9995%) with a flow rate of 1 mL/min, with no splitting. The initial column temperature was 40 °C, maintained for 5 min, then increased to 80 °C at a rate of 2 °C/min, next increased to 150 °C at a rate of 5 °C/min, and finally increased to 250 °C at a rate of 10 °C/min, maintained for 5 min. Mass spectrometry electron bombardment ion source (EI) temperature: 280 °C/Electron energy 70 eV; Scan range: 30–450 amu.

Data processing: The collected mass spectrograms were searched in the NIST08 Mass Spectral Library, and each chromatographic peak was semi-quantified by the internal standard method.

### 2.9. Detection of “Four Dominant Acids”

The “four dominant acids” (L-lactic acid, acetate, butyrate and caproate) in fermentation samples were detected by high performance liquid chromatography (HPLC) and quantified by external standard method.

Sample treatment: The liquid fermented samples were centrifuged at 4 °C 10,000 rpm for 3 min, supernatants were filtered with 0.22 μm membrane and sealed in the sample bottle. The solid-state fermentation sample was weighed at 2.00 g and 8 mL of double distilled water was added, after ultrasonic extraction for 60 min in 40 °C water bath, the mixtures were centrifuged at 3000 rpm for 5 min, then the supernatants were collected and filtered with 0.22 μm membrane before sealing in the sample bottle.

Chromatography conditions: An Agilent 1260 Infinity II instrument (Agilent Technologies, Inc., Santa Clara, CA, USA) was used for HPLC detection. The separation was performed on a Poroshell 120 EC-C18 (4.6 mm × 150 mm, 4 μm) column using 0.5% (*w*/*v*) NaH_2_PO_4_ as mobile phase. The injection volume was 20 μL; Flow rate was 0.25 mL/min for 0–10 min and 1 mL/min for 10–15 min; Column temperature was 40 °C and UV detection wavelength was 210 nm.

### 2.10. Statistical Analysis

Significance analysis of RT-qPCR, GC-MS and HPLC datum were performed using SPSS (Version 24, IBM, Armonk, NY, USA) based on ANOVA test with LSD and Waller-Duncan hypothesis (*p* < 0.05), the results were presented as the mean ± standard deviation (S.D.). Redundancy analysis (RDA) was conducted and visualized by Canoco 5.0 (Wageningen, The Netherlands). Principal co-ordinates analysis (PCoA) based on Bray-Curtis distance were performed by MetaboAnalyst 5.0 (http://www.metaboanalyst.ca/, accessed on 18 August 2021), and heatmap were performed using R 3.6.1 (Vienna, Austria).

## 3. Results

### 3.1. Screening Functional Microorganisms

By annotating the sequence information of gene *ME2* in NCBI, *Ruminococcaceae bacterium* CPB6 which is prevalent in pit mud, especially in aged pit mud [4,21], has the highest similarity. Its 16S rRNA sequence information was input into GRPWREC to predict the optimal screening medium, and the result showed that the optimal medium was DSMZ_330. By species annotation of gene *adhE* in NCBI, *Eubacterium limosum* 81C1 has the highest similarity, which together with *Ruminococcaceae bacterium* CPB6 both belong to the class Clostrida, indicating that the gene expression of Clostrida is very active in the aged pit mud. The predicted optimal medium for *Eubacterium limosum* 81C1 screening was DSMZ_614.

DSMZ_330 liquid medium was used for anaerobic enrichment and cultivation of the gene *ME2*-containing microorganisms in aged pit mud. The enrichment cultures were coated on DSMZ_330 solid plate medium for anaerobic cultivation until the colonies were grown. Colonies (48) with morphological characteristics of bacterial colonies were randomly selected and colony PCR was conducted, among them, eight colonies had correct PCR amplification bands, and their serial numbers were 3, 4, 5, 7, 29, 30, 31 and 42, respectively. Their agarose gel electrophoresis images are shown in Figure 1C. The amplification bands shown in Figure 1C were cut and purified, the result of paired-end sequencing of the purified products were aligned in NCBI by BLASTN, and the results showed that all the products were from genes *ME2*, indicating that these eight strains were all gene *ME2-* containing strains. These eight strains were expansion cultured, then the cells were collected by centrifugation at 4 °C and 10,000 rpm for 3 min, and their 16S rRNA genes were sequenced for species identification. The results showed that strains 3, 4, 5, 7, 29 and 30 were not pure, while strains 31 and 42 were *Rummeliibacillus suwonensis*. They were named *Rummeliibacillus suwonensis* M31(RsM31) and *Rummeliibacillus suwonensis* M42(RsM42).

The same method was used to screen the gene *adhE*-containing microorganisms in aged pit mud with DSMZ_614 medium. Similarly, 48 colonies were selected for PCR, among which colonies 1, 2, 5, 6, 7, 8, 9 and 12 had correct amplification bands (Figure 1D). The correct bands were cut, purified and sequenced, the results showed that all the products were from *adhE* genes. Then these strains were expansion cultured, and their 16S rRNA were sequenced, the results showed that strain 6 was *Clostridium tyrobutyricum* and strain 7 was *Lactobacillus buchneri*, they were named *Clostridium tyrobutyricum* A6 (CtA6) and *Lactobacillus buchneri* A7 (LbA7). Other strains were not pure.

Phylogenetic trees were constructed by neighbor joining (NJ) based on the 16S rRNA gene information of each strain (Figure 1E). It could be seen that the three species screened from aged pit mud were not close in terms of genetic relationship. The two gene *adhE* contained strains, CtA6 and LbA7, had a distant genetic relationship, in addition, they were remotely related to *Eubacterium limosum*, which was the species aligned in NCBI according to the sequence of gene *adhE*, suggesting that different microorganisms in LFB brewing microbial community may have common functions. Similarly, the screened strain *Rummeliibacillus suwonensis* which containing gene *ME2* also has a distant genetic relationship with the strain *Ruminococcaceae bacterium* CPB6 which was the species aligned in NCBI according to the sequence of gene *ME2*.

### 3.2. Liquid Fermentation of Functional Microorganisms

Functional microorganisms RsM31, RsM42, CtA6 and LbA7 were inoculated into liquid fermentation medium for simulated fermentation. Firstly, the trends of the change of expression quantities of the key genes (*ME2* and *adhE*) during fermentation was analyzed (Table 3). As the fermentation progressed, the expression quantities of genes *ME2* in RsM31 and RsM42 decreased gradually, and the changes at each stage were significant (*p* < 0.05), but there were differences in the decrease range and rate of key gene expression quantities between the two functional strains during the whole fermentation period. The gene *ME2* expression quantity in RsM31 decreased by 38.9% from day 0 to day 27, and its decrease range at each fermentation stage was greater than that in RsM42, the expression quantity of gene *ME2* in RsM42 decreased only 7.2% from day 0 to day 27, indicating that there was a great difference of gene *ME2* expression quantity between the two strains during liquid fermentation. Strains CtA6 and LbA7 also showed a similar trend of expression quantities changes of key gene during the liquid fermentation process. The expression quantities of gene *adhE* decreased in general during the fermentation process, especially the expression changes from day 0 to day 13 were significant (*p* < 0.05), the quantities of gene *adhE* expression were basically stable on the 13th day of fermentation, and there was no significant change during the followong fermentation (*p* > 0.05). In terms of the range and rate of the decrease of gene *adhE* expression quantities, during the fermentation process, strains CtA6 and LbA7 showed similar performance, indicating that there was basically no difference in the expression quantity of key genes during the liquid fermentation process.

**Figure 2:**
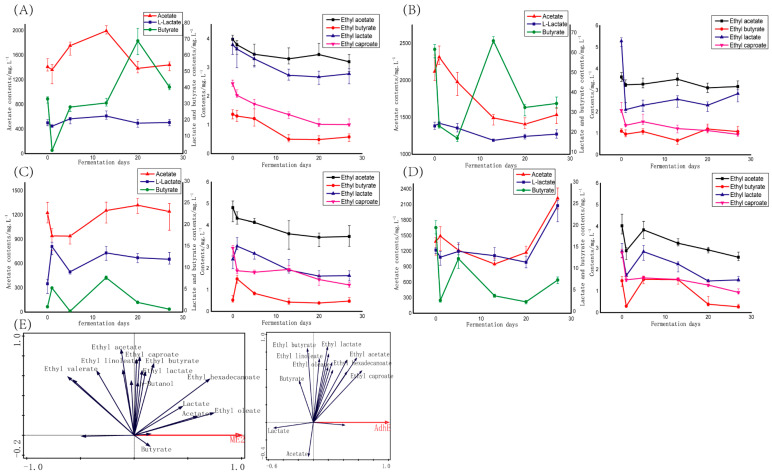
Effects of functional microorganisms on major flavor substances during liquid fermentation. (**A**–**D**) were changes of key flavor substances during liquid fermentation after RsM31, RsM42, CtA6 and LbA7 inoculation, respectively. (**E**) was redundancy analysis of gene *ME2*, *adhE* and main flavor substances.

During the liquid fermentation process of the four functional microorganisms, a total of 42 flavor substances were identified, among which esters were the main substances, followed by alcohols, aldehydes, acids and a small amount of heterocyclic compounds, and the main flavors were analyzed (Tables S2 and S3 and Figure 2). As shown in Figure 2A, during the liquid fermentation process of RsM31, from day 1 the three main organic acids generally presented the trend of rising during the early stage and falling during the late stage of fermentation. From day 1 to day 13 the gene *ME2* expression quantity was still at a high level and the corresponding enzyme quantity is also relatively sufficient, which may be the reason why the organic acid content increased, while the possible reason for the decrease of organic acid content in the late stage was that the gene *ME2* expressed by RsM31 decreased and the corresponding enzyme decreased with the extension of fermentation time. During the fermentation process of RsM31, the contents of the “four dominant esters” gradually decreased, and the decline rate was relatively faster during the first 13 days, and after 13 days of fermentation, the contents of the four major esters were basically in balance, which was consistent with the changes of gene *ME2* expression quantities analyzed above. In the early stage, due to the high expression level of gene *ME2* and the corresponding enzyme, the content of the “four dominant esters” decreased rapidly, in the later stage, due to the gradual decline of gene *ME2* and the corresponding enzyme, the content of the “four dominant esters” tended to be stable. According to Figure 2B, the change trend of organic acids and the “four dominant esters” in the liquid fermentation of RsM42 was similar to that in the fermentation process of RsM31.

Next, the effects of gene *adhE*-containing functional microorganisms CtA6 and LbA7 on organic acids and “four dominant esters” during liquid fermentation were analyzed. As can be seen from Figure 2C, the variation trends of the three organic acids in liquid fermentation of CtA6 were different to some extent. Acetate content decreased rapidly after inoculation, then gradually increased after the 5th day of fermentation, and leveled off after the 13th day of fermentation. In the early fermentation stage, a large quantity of gene *adhE* was expressed and the corresponding enzyme is sufficient, maybe leading to the decreased acetate content, in the middle stage, the expression quantity of gene *adhE* and the corresponding enzyme decreased, the acetate content began to increase. In the late stage, the expression of gene *adhE* and the corresponding enzyme tended to be stable, the acetate content also reached a balance. The contents of butyrate and lactate increased rapidly after inoculation, then gradually decreased from the 13th day and finally stabilized. These trends were quite different from the influence of gene *ME2*. In CtA6 fermentation process, the content of ethyl acetate and ethyl lactate gradually declined and finally leveled off, which was consistent with the change trend of gene *adhE* expression quantity during the fermentation process. The contents of ethyl butyrate and ethyl lactate increased after CtA6 inoculation, and then gradually decreased from day 1 and finally stabilized. Although LbA7 and CtA6 were both functional microorganisms screened based on gene *adhE*, but they are different species, so their effects on flavor substances in fermentation were different to some extent. From the variation trend of organic acids in the LbA7 fermentation process (Figure 2D), after a slow decline in the early and middle stages, the contents of the three organic acids all showed a significant increase in the late fermentation period. From the content changes of the “four dominant esters” (Figure 2D), after LbA7 inoculation, all four esters decreased rapidly, and then increased rapidly from the first day to the 15th day of fermentation, then gradually decreased and finally stabilized. This was basically consistent with the change trend of gene *adhE* expression quantity of LbA7 during fermentation.

Redundancy analysis (RDA) was used to further analyze the correlation between key genes *ME2*/*adhE* and major flavor substances during liquid fermentation of functional microorganisms (RsM31, RsM42, CtA6 and LbA7). As shown in Figure 2E, the gene *ME2* was positively correlated with butyrate, acetate, lactate, as well as higher fatty acid levels, which further confirmed that the role of gene *ME2* in LFB production was to promote the synthesis of organic acids and ethyl fatty acids. Gene *ME2* also had a certain positive correlation with the important esters in LFB, such as ethyl caproate, ethyl butyrate and ethyl lactate, but the correlation is not as strong as that of organic acids. There was a strong positive correlation between the gene *adhE* and ethyl caproate, ethyl valerate and ethyl acetate, indicating that gene *adhE*-containing strains may promote the synthesis of ethyl caproate and so on, which are important flavor compounds in LFB, so it is beneficial to improve the quality of LFB. Gene *adhE* was negatively correlated with organic acids such as butyrate, lactate and acetate, which might be because gene *adhE* was positively correlated with esters synthesis, while organic acids are substrates of ester synthesis, so there was a significant negative correlation between gene *adhE* and organic aicds. Taken together, RsM31 and RsM42 could promote the synthesis of organic acids, and organic acid as the substrate under the action of CtA6 and LbA7 could synthesis esters, especially the “four dominant esters” in LFB, therefore the cooperation of the two kinds of microorganisms plays an important role in improving the quality of LFB.

### 3.3. Solid Fermentation of Functional Microorganisms

The effect of microorganisms containing genes *ME2* and *adhE* were found through liquid fermentation. However, LFB was produced through solid fermentation, in order to study the role of functional microorganisms RsM31, RsM42, CtA6 and LbA7 in solid fermentation, they were inoculated into *zaopei* at different inoculum sizes, and simulated fermentation were carried out under laboratory conditions to analyze the composition structure change trend of major flavor substances.

**Figure 3:**
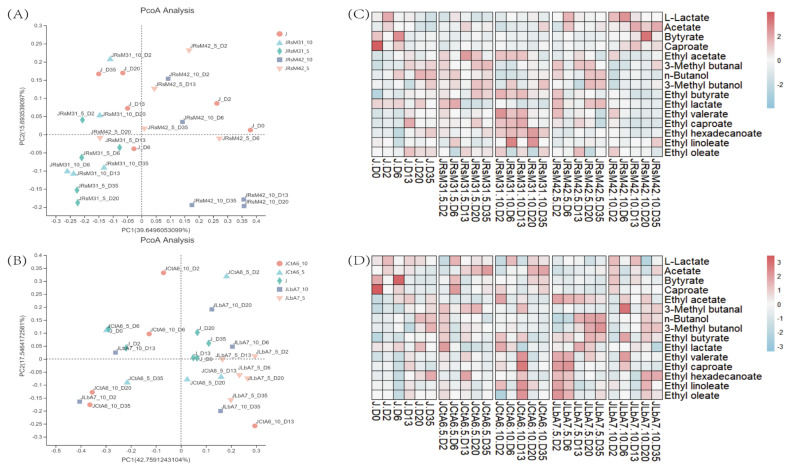
Effect of functional microorganisms on major flavor substances during liquid fermentation. Figure (**A**,**B**) were principal co-ordinates analysis of RsM31, RsM42, CtA6 and LbA7 fermented in zaopei, in the figures J represent zaopei without inoculation; “5 and 10” meant 5% and 10% inoculation amount during fermentation; “D0, D2, D6, D13, D20, D35” represent fermented days. Figure (**C**,**D**) were heat map of flavor substances in solid fermentation of functional microorganisms inoculated zaopei, in the figures the redder the boxes, the higher the substances content, and the bluer the boxes, the lower the substances content.

Firstly, principal co-ordinates analysis (PCoA) was used to analyze the structure difference of flavor compounds composition in the *zaopei* fermented by four functional microorganisms. As shown in Figure 3A, the compositional structure of flavor substances of RsM31 and RsM42-fermented *zaopei* were significantly different, and after inoculation with RsM31, the samples were distributed on the left side of the central axis during the whole fermentation process, while the samples inoculated with RsM42 were basically distributed on the right side of the central axis, indicating that the two strains had significant differences in fermentation. In terms of the effect of inoculation amount, there were some differences between the two strains, and the inoculation amount had a more obvious effect on RsM42, especially in the middle and late fermentation stage (day 13, day 20 and day 35). The samples inoculated with 10% amount of RsM42 were all concentrated in the lower right part of the axis, far away from the group. From the perspective of whether or not the *zaopei* were inoculated, it could be seen that they were differences, and the difference increased with the extension of fermentation time, indicating that both inoculated RsM31 and RsM42 had significant changes in the composition structure of flavor substances during fermentation.

The effects of CtA6 and LbA7 for *zaopei* fermentation showed that the samples of uninoculated *zaopei* at each fermentation stage were concentrated around the center of the coordinate axis, while the samples after inoculation were distributed around the center, indicating that strains CtA6 and LbA7 had significant influence on the composition structure of flavor substances in *zaopei* (Figure 3B). There were also some differences between strains CtA6 and LbA7 in the fermentation process. Most of the samples fermented by CtA6 were distributed on the left side of the central axis, while most of the samples after fermentation of LbA7 were distributed on the right side, indicating that these two strains had different effects on the fermentation process of *zaopei*. From the effects of inoculation amount on fermentation, the samples inoculated with 5% LbA7 were closer to the center of the axis, while the samples inoculated with 10% LbA7 were further dispersed, this indicated that when the inoculation amount of LbA7 was small there was little difference to uninoculated *zaopei*, and increasing the inoculation amount would increase the difference in the fermentation process, however, when the fermentation time reached to 35 days the difference in inoculation amount is no longer significant. Moreover, there was also a great difference between inoculated CtA6 at 5% and 10% during fermentation, and the difference was no longer obvious after fermentation time reached to 35 days. The results showed that no matter how much inoculated the two strains were, there might be differences in the fermentation process, but when the fermentation time was long enough, there was little difference in the composition structure of the final flavor substances.

In order to further analyze the difference of main flavor substances during the fermentation of functional microorganisms, the content of flavor substances in different fermentation stages were displayed by heat map. From the changes of main flavor substances caused by RsM31 and RsM42 inoculation (Figure 3C), after RsM31 inoculation, the contents of the main acids decreased in the early fermentation period compared with the uninoculated *zaopei*, but the contents of acetic acid increased in the late fermentation period, while the contents of other three organic acids had no difference with uninoculated *zaopei*. After RsM42 inoculation, the contents of the main acids in *zaopei* increased significantly compared with those in the *zaopei* without inoculation, especially when the inoculation amount reached 10%. This change fully indicated that the functional microorganisms screened based on gene *ME2*, especially RsM42, has a significant role in promoting the synthesis of organic acids. From the perspective of the changes of the “four dominant esters”, after inoculation of RsM31, the quantities of synthesized ethyl acetate, ethyl butyrate and ethyl caproate increased significantly relative to the uninoculated *zaopei*, especially in the early stage of the fermentation, while the change trend of ethyl lactate was the opposite, and it obviously decreased relative to uninoculated *zaopei*, so inoculation of RsM31 was beneficial to the quality of LFB, and the larger the amount of RsM31 inoculation, the more the relative range of “four dominant esters” changed. The changes of the “four dominant esters” in *zaopei* after RsM42 inoculation were not obvious compared with those in *zaopei* without inoculation. Only in *zaopei* inoculated with 5% RsM42, the synthesis of ethyl lactate increased slightly during the late fermentation period. Therefore, in general, RsM31 was more conducive to the synthesis of esters in *zaopei*, while RsM42 was more conducive to the synthesis of organic acids.

From the content changes of the “four dominant acids” (Figure 3D), it is shown that after CtA6 inoculation the contents of the main acids increased significantly during the later fermentation period compared with the uninoculated *zaopei*, and the higher the amount of CtA6 inoculation, the more contents of the main acids increased. In the *zaopei* inoculated with LbA7, the contents of the “four dominant acids” were lower than those in the uninoculated *zaopei* at the early stage of fermentation, and basically equal to those in the uninoculated *zaopei* at the late stage of fermentation. In general, the contents of the “four dominant acids” in the *zaopei* inoculated with larger LbA7 inoculation were slightly higher. Analyzing the changes of the “four dominant esters”, it was showed that after inoculation of CtA6 relative to uninoculated *zaopei*, the content of ethyl acetate during the late fermentation fell slightly, ethyl butyrate and ethyl lactate relatively rose during the early stage of the fermentation and fell slightly during the late stage, and the ethyl caproate content changed little. In general, the “four dominant esters” contents rose with the increase of the inoculation amount of CtA6. The yield of the “four dominant esters” in *zaopei* inoculated with LbA7 was higher than in that inoculated with CtA6. The contents of ethyl acetate, ethyl butyrate and ethyl caproate were higher than those of uninoculated *zaopei* during the whole fermentation process, while the contents of ethyl lactate displayed little change, and even decreased during the late fermentation stage. This change trend was also beneficial to the improvement of LFB quality.

## 4. Discussion

The sequence of gene *ME2* was annotated in NCBI, and the most similar species was *Ruminococcaceae bacterium* CPB6, which has the function of synthesizing caproic acid using lactic acid as substrate [3,22], a function that is beneficial to the quality of LFB. Therefore, it was speculated that the gene *ME2* may be related to the function of increasing caproic acid and decreasing lactic acid. However, the two strains containing gene *ME2* we have screened were *Rummeliibacillus suwonensis*, which has been reported screened from soil [23] and vinegar grains [24], it was reported that *Rummeliibacillus suwonensis* was thermophilic and simultaneous anaerobic, and could tolerate higher concentrations of ethanol (8%, vol) and salt (13%, wt/vol) [24], therefore, the bacterium was suitable for survival in the relatively harsh LFB fermentation environment. This study confirmed that not only *Ruminococcaceae bacterium* CPB6 but also *Rummeliibacillus* contained gene *ME2* in the microbial community of LFB fermentation. The most similar species annotated by sequence of gene *adhE* in NCBI was *Eubacterium limosum*, which has the ability to change acetic acid into butyric acid [25]. The gene *adhE* was an important functional gene expressed by *Eubacterium limosum*, and it has been confirmed that the gene *adhE* was related to butyric acid metabolism [18], therefore, the gene *adhE* may have an important influence on the formation of butyric acid in LFB brewing process, and further affect the composition and structure of flavor substances in LFB. However, the two strains containing gene *adhE* we have screened were *Clostridium tyrobutyricum* and *Lactobacillus buchneri*. *Clostridium tyrobutyricum* has been isolated from the fermentation products such as milk [26], silage [27] and cheese [28], and like *Eubacterium limosum* both of them belong to Clostridia [29], and also have the ability to synthesize butyric acid [30], indicating that these two strains may have similar functions. Therefore, *Clostridium tyrobutyricum* may have an important impact on LFB production. *Lactobacillus buchneri* also have been isolated from silage [31] and fermented food [32]. This bacterium had a good tolerance to ethanol [33], indicating that it was suitable for playing its role in the LFB brewing environment. Studies have reported that lactic acid content will decrease and the contents of acetic acid, ethanol and ethyl acetate will increase after the fermentation of *Lactobacillus buchneri* [32], which will have an important impact on the flavor of LFB. Therefore, *Lactobacillus buchneri* was also an important functional microorganism in the fermentation process of LFB. This study confirmed that the key genes in LFB fermentation process were expressed by different microorganisms, and the function of brewing microbial community was not completed by some single microorganisms, but by the joint action of many microorganisms.

In order to verify whether the selected functional microorganisms with high expression of key genes were effective in the fermentation process of LFB, in this study, strains RsM31 and RsM42 screened based on gene *ME2*, and strains CtA6 and LbA7 screened based on gene *adhE* were used to simulate LFB brewing by the liquid culture method. Then the changes of expression levels of the two key genes and the composition and structure of flavor substances were explored to investigate the effect mechanism of genes *ME2* and *adhE*. With the progress of fermentation, the expression levels of the two key genes decreased to varying degrees, indicating that the changes in the composition of the medium in the fermentation process may inhibit the expression of the two key genes. From the regularity of flavor component changes in fermentation process, the expression of gene *ME2* in strains RsM31 and RsM42 was positively correlated with the contents of the main acids, which fully proved that the expression of gene *ME2* could promote the synthesis of acids. It can be seen from Figure 1A that the expression of gene *ME2* can catalyze the production of pyruvate from malic acid and oxaloacetic acid, and we have proved that pyruvate was an important substrate for the synthesis of the “four dominant acids” [13], which may be an important reason why the expression of gene *ME2* can promote the synthesis of the main acids. The effects of strains CtA6 and LbA7 on the main acids were different from RsM31 and RsM42, the expression level of gene *adhE* was negatively correlated with the contents of the main acids. Studies have confirmed that the expression level of gene *adhE* can promote the acetyl-CoA and butanoyl-CoA to synthesize ethanol, butanol and other alcohols, thus reducing the probability of organic acid synthesis [18]. This can also be confirmed by the correlation analysis between gene *adhE* expression level and butanol content in this study, therefore, the expression of gene *adhE* in strains CtA6 and LbA7 resulted in the decrease of organic acid content in liquid simulated fermentation. From the perspective of the changes of the “four dominant esters”, gene *adhE* was significantly positively correlated with the contents of the “four dominant esters”, while the correlation with gene *ME2* was not strong. The results of liquid fermentation research indicated that the two key genes played different roles in LFB brewing process, the high expression of gene *ME2* could promote the production of the main acids, and under the action of gene *adhE*, the main acids can be used as substrates to produce the “four dominant esters”. The two key genes complement each other, which played an important role in promoting the formation of key flavor substances in LFB and was very beneficial to improve the quality of LFB.

In order to further verify the roles of the two key genes during LFB brewing, the functional microorganisms were inoculated into *zaopei* and fermented with a method closer to the actual production of LFB, then their effects on the composition and structure of major flavor substances were analyzed. The contents of the “four dominant acids” in *zaopei* increased significantly after the inoculation of RsM31 and RsM42, suggesting that the mechanism of action of gene *ME2* discovered through liquid fermentation was also applicable in the solid fermentation process. During the fermentation of RsM31 and RsM42 inoculated *zaopei* the contents of the “four dominant esters” relatively increased except for ethyl lactate, so in general, the two functional strains screened based on gene *ME2* not only can promote the synthesis of the “four dominant acids” and had the ability to use the “four dominant acids” as substrates to promote the beneficial esters synthesis. This is in conformity with the law of LFB brewing, and during fermentation, ethyl caproate, ethyl butyrate and ethyl acetate increased more obviously, the ethyl lactate was slightly down, that was great for the quality of LFB, which fully showed that RsM31 and RsM42 strengthening of LFB brewing had the vital significance, can obviously improve the quality of LFB. The fermentation of strains CtA6 and LbA7 screened based on gene *adhE* in *zaopei* had different effects on the contents of the “four dominant acids”. The fermentation of CtA6 in *zaopei* promoted the generation of the main acids, especially during the late fermentation period. While the fermentation of LbA7 in *zaopei* had little effect on the contents of the “four dominant acids”, in the early fermentation stage, the contents of “four dominant acids” were even lower than those of uninoculated *zaopei*. By and large, the effects of the two functional strains on the “four dominant esters” were that same, as both could promote the generation of ethyl caproate, ethyl butyrate and ethyl acetate, which also confirmed the promotion effect of gene *adhE* on ester production, and it was also beneficial to the quality of LFB.

## 5. Conclusions

In this study, two functional strains *Rummeliibacillus suwonensis* (RsM31 and RsM42) were screened based on information of gene *ME2*, and the functional microorganisms *Clostridium tyrobutyricum* (CtA6) and *Lactobacillus Buchneri* (LbA7) were screened based on information of gene *adhE.* Then it was confirmed that the function of microbial community in LFB brewing was not accomplished by a single microorganism, but rather due to a combination of microbes. The mechanism of action of the two key genes was quite different, the key gene *ME2* was significant positive correlate the contents of the main acids in LFB brewing process, however the key gene *adhE* and the formation of the main esters in LFB brewing process was a significant positive correlation. The two key genes complement each other in LFB brewing process playing an important role in promoting the formation of flavor substances, and are very beneficial to improving the quality of LFB.

## Figures and Tables

**Table 1:** Colony PCR reaction system and procedure.

Gene	Reagent	Volume of Addition	Primer	PCR Procedure
*ME2*	2 × T5 Super PCR Mix (Colony) (Tsingke Biotechnology Co., Ltd.)	12.5 µL	Forward Primer:(*ME2*-F): 5′-CTATTGCGAAGCACCTG-3′ Reverse Primer (*ME2*-R): 5′-AAACTCCCCTGTTTATGTT-3′ Product length: 148 bp	Initial denaturation at 98 °C for 180 s; followed by 32 cycles of denaturation at 98 °C for 10 s, annealing at 49 °C for 30 s and elongation at 72 °C for 30 s; then final elongation at 72 °C for 120 s.
	Forward Primer (*ME2*-F, 10 µM)	1 µL		
	Reverse Primer (*ME2*-R, 10 µM)	1 µL		
	Template	2 µL		
	ddH_2_O up to	25 µL		
*adhE*	2 × T5 Super PCR Mix (Colony) (Tsingke Biotechnology Co., Ltd.)	12.5 µL	Forward Primer (*adhE*-F): 5-GATGCTTTGATTGCCCTTGG-3′ Reverse Primer (*adhE*-R): 5-AAACGGGTTGTTGTTGGTG-3′ Product length: 354 bp	Initial denaturation at 98 °C for 180 s; followed by 32 cycles of denaturation at 98 °C for 10 s, annealing at 58 °C for 30 s and elongation at 72 °C for 30 s; then final elongation at 72 °C for 120 s.
	Forward Primer (*adhE*-F, 10 µM)	1 µL		
	Reverse Primer (*adhE*-R, 10 µM)	1 µL		
	Template	2 µL		
	ddH_2_O up to	25 µL		

**Table 2:** Primers for qPCR of gene *ME2* and *adhE*.

Gene	Primer	Sequence 5’-3’	Annealing Temperature	Products Length
*adhE*	*adhE*-2F	GTATTCCAAATGTCAGCG	48.6 °C	73 bp
	*adhE*-2R	TTGATTTCTTTACAGAGGGT	49.0 °C	
*ME2*	*ME2*-F	CTATTGCGAAGCACCTG	49.6 °C	148 bp
	*ME2*-R	AAACTCCCCTGTTTATGTT	48.6 °C	

**Table 3:** Expression quantities of key genes of functional strains in different liquid fermentation stages.

Genes	Strains	Key Genes Expression Quantities (log10 Copies/mL)					
		0 d	1 d	5 d	13 d	20 d	27 d
*ME2*	RsM31	5.96 ± 0.02 ^f^	5.50 ± 0.02 ^g^	4.58 ± 0.02 ^h^	4.13 ± 0.02 ^i^	3.84 ± 0.02 ^j^	3.64 ± 0.01 ^k^
	RsM42	6.90 ± 0.01 ^a^	6.86 ± 0.01 ^ab^	6.81 ± 0.01 ^b^	6.72 ± 0.01 ^c^	6.64 ± 0.01 ^d^	6.50 ± 0.01 ^e^
*adhE*	CtA6	12.30 ± 0.03 ^a^	11.44 ± 0.02 ^b^	11.33 ± 0.01 ^c^	11.22 ± 0.02 ^d^	11.21 ± 0.01 ^d^	11.20 ± 0.01 ^d^
	LbA7	11.48 ± 0.01 ^b^	11.09 ± 0.01 ^e^	10.99 ± 0.01 ^f^	10.93 ± 0.01 ^g^	10.91 ± 0.01 ^g^	10.91 ± 0.01 ^g^

Note: Different letters behind the figures indicated significant differences (*p* < 0.05).

## Data Availability

The data that support the findings of this study are available on request from the corresponding author. The data are not publicly available due to privacy or ethical restrictions.

## Supplementary Materials

The following supporting information can be downloaded at: www.mdpi.com/article/10.3390/foods11050700/s1, Table S1: The preparation method of DSMZ_330 and DSMZ_614; Table S2: Contents of flavor substances produced by RsM31and RsM42 at different liquid fermentation stages; Table S3: Contents of flavor substances produced by CtA6 and LbA7 at different liquid fermentation stages.
