# Supplementary material for: Study on the Effect of Key Genes ME2 and adhE during Luzhou-Flavor Baijiu Brewing
Source: Foods. 2022 Feb 26;11(5):700. doi: 10.3390/foods11050700 (PMC8909148; doi:10.3390/foods11050700)
Supplement: Supplementary file 1 [file foods-11-00700-s001.zip › foods-1589793-supplementary/Table S1.pdf]

Table S1. The preparation method of DSMZ_330 and DSMZ_614.

Medium	Recipe	Collocation Method	Supplementary Instruction
DSMZ_330<br>RUMEN BACTERIA MEDIUM	Mineral solution 38.00 mL;<br>K_2HPO_4 0.30 g;<br>Trypticase peptone (BD BBL) 2.00 g;<br>Yeast extract (OXOID) 0.50 g;<br>Volatile fatty acid mixture 3.10 mL;<br>Haemin solution (0.05% w/v) 2.00 mL;<br>Glycerol 0.50 g;<br>Na-resazurin solution (0.1% w/v) 0.50 mL;<br>Na_2CO_3 4.00 g;<br>D-Glucose 0.50 g;<br>Maltose 0.50 g;<br>Cellobiose 0.50 g;<br>Starch (soluble) 0.50 g;<br>L-Cysteine-HCl × H_2O 0.25 g;	Dissolve ingredients (except carbonate, glucose, maltose, cellobiose, soluble starch, cysteine and sulfide), then sparge medium with 100% CO_2 gas for 30-45 min to make it anoxic. Add the carbonate and equilibrate the medium with the CO_2 gas to pH 6.8.<br>Distribute under 100% CO_2 gas atmosphere into anoxic Hungate-type tubes or serum vials and autoclave. Thereafter, add glucose, maltose, cellobiose, soluble starch, cysteine and sulfide from sterile anoxic stock solutions prepared under 100% N_2 gas atmosphere.<br>Adjust pH of complete medium to 6.7-6.8, if necessary.	Mineral solution:<br>KH_2PO_4 6.00 g;<br>NaCl 12.00 g;<br>$(NH_4)_2SO_4$ 6.00 g;<br>$CaCl_2$ ×2 H_2O 1.60 g ;<br>$MgSO_4$ × 7 H_2O 2.50 g;<br>Distilled water 1000.00 mL.<br><br>Volatile fatty acid mixture:<br>Acetic acid 548.50 mL;<br>Propionic acid 193.50 mL;<br>Butyric acid 129.00 mL;<br>n-Valeric acid 32.25 mL;<br>iso-Butyric acid 32.25 mL;

	$Na_2S \times 9\ H_2O$ 0.25 g;<br>Distilled water 960.00 mL.		DL-2-Methyl butyric acid 32.25 mL;<br>iso-Valeric acid 32.25 mL.
DSMZ_614<br>ACETOBACTERIUM FIMETARIUM MEDIUM	KCl 0.33 g;<br>$MgCl_2 \times 6\ H_2O$ 0.52 g ;<br>$CaCl_2 \times 2\ H_2O$ 0.22 g;<br>NH_4Cl 0.33 g;<br>KH_2PO_4 0.33 g;<br>Yeast extract 0.50 g;<br>Trace element solution 10.00 mL;<br>Na-resazurin solution (0.1% w/v) 0.50 mL;<br>$NaHCO_3$ 1.00 g;<br>Vitamin solution 10.00 mL;<br>D-Fructose 10.00 g;<br>$Na_2S \times 9\ H_2O$ 0.70 g;<br>Distilled water 1000.00 mL.	Dissolve ingredients (except bicarbonate, vitamins, fructose and sulfide) and sparge medium with 100% N_2 gas for 30-45 min to make it anoxic. Dispense medium under same gas atmosphere into anoxic Hungate-type tubes or serum vials and autoclave. Before use add vitamins, fructose and sulfide from sterile anoxic stock solutions prepared under 100% N_2 gas and bicarbonate from a sterile anoxic stock solution prepared under 80% N_2 and 20% CO_2 gas atmosphere. Vitamins should be sterilized by filtration. Adjust pH of complete medium to 6.8-7.0, if necessary.	Trace element solution:<br>Nitrilotriacetic acid 1.50 g;<br>$MgSO_4 \times 7\ H_2O$ 3.00 g;<br>$MnSO_4 \times H_2O$ 0.50 g;<br>NaCl 1.00 g;<br>$FeSO_4 \times 7\ H_2O$ 0.10 g;<br>$CoSO_4 \times 7\ H_2O$ 0.18 g;<br>$CaCl_2 \times 2\ H_2O$ 0.10 g;<br>$ZnSO_4 \times 7\ H_2O$ 0.18 g;<br>$CuSO_4 \times 5\ H_2O$ 0.01 g;<br>$KAl(SO_4)_2 \times 12\ H_2O$ 0.02 g;<br>H_3BO_3 0.01 g;<br>$Na_2MoO_4 \times 2\ H_2O$ 0.01 g;<br>$NiCl_2 \times 6\ H_2O$ 0.03 g;

$Na_2SeO_3 \times 5\ H_2O$ 0.30 mg;

$Na_2WO_4 \times 2\ H_2O$ 0.40 mg;

Distilled water 1000.00 mL;

First dissolve nitrilotriacetic acid and adjust pH to 6.5 with KOH, then add minerals.

Adjust final to pH 7.0 with KOH.

Vitamin solution:

Biotin 2.00 mg;

Folic acid 2.00 mg;

Pyridoxine-HCl 10.00 mg;

Thiamine-HCl 5.00 mg;

Riboflavin 5.00 mg;

Nicotinic acid 5.00 mg;

D-Ca-pantothenate 5.00 mg;

Vitamin B12 0.10 mg;

	p-Aminobenzoic acid 5.00 mg;<br>Lipoic acid 5.00 mg;<br>Distilled water 1000.00 mL.
