# Supplementary material for: Study on the Effect of Key Genes ME2 and adhE during Luzhou-Flavor Baijiu Brewing
Source: Foods. 2022 Feb 26;11(5):700. doi: 10.3390/foods11050700 (PMC8909148; doi:10.3390/foods11050700)
Supplement: Supplementary file 1 [file foods-11-00700-s001.zip › foods-1589793-supplementary/Table S2.pdf]

Table S2. Flavor substances contents produced by RsM31and RsM42 in different liquid fermentation stages.

Flavor substances（mg/L）	K	RsM31-0	RsM31-1	RsM31-5	RsM31-13	RsM31-20	RsM31-27	RsM42-0	RsM42-1	RsM42-5	RsM42-13	RsM42-20	RsM42-27
L-Lactic acid	13.42±0.91	19.14±1.12	17.15±0.38	20.81±1.21	23.67±1.50	18.82±1.41	19.26±1.16	24.27±1.05	25.47±0.55	23.25±1.23	17.12±0.21	18.93±0.70	20.23±1.25
Acetic acid	1353.37±60.97	1431.78±59.01	1313.71±92.34	1721.82±63.81	2005.14±39.36	1398.46±54.18	1425.31±42.16	2154.43±101.62	2331.35±72.05	1958.45±88.63	1471.82±37.15	1415.46±43.25	1544.72±60.82
Butyric acid	22.76±1.49	33.41±1.13	2.11±0.16	28.03±0.95	31.68±1.39	69.50±4.68	41.25±1.06	62.10±1.95	24.34±0.73	17.94±0.84	67.31±0.97	32.66±1.73	35.81±1.62
2-Methylfuran	0.02±0.01	0.06±0.01	0.06±0.00	0.07±0.00	0.08±0.00	0.08±0.01	0.10±0.01	0.05±0.00	--	0.07±0.00	0.07±0.01	0.05±0.01	0.10±0.01
Ethyl acetate	3.45±0.30	3.84±0.20	3.73±0.14	3.44±0.22	3.37±0.17	3.47±0.13	3.17±0.17	3.61±0.12	3.30±0.10	3.32±0.13	3.50±0.16	3.14±0.16	3.21±0.12
3-Methyl butanal	0.08±0.04	0.08±0.01	0.09±0.02	0.08±0.01	--	0.07±0.01	--	0.07±0.01	0.07±0.01	0.04±0.00	0.07±0.01	--	--
n-Butanol	0.84±0.0	1.00±0.1	0.94±0.1	0.81±0.0	0.86±0.0	0.73±0.0	0.86±0.0	0.83±0.0	0.77±0.0	0.80±0.0	0.92±0.0	1.04±0.1	0.72±0.1

	5	2	0	7	7	4	5	8	6	8	6	0	0
3-Methyl butanol	0.17±0.04	0.16±0.01	0.16±0.01	0.19±0.03	0.18±0.02	0.18±0.02	0.20±0.03	0.17±0.03	0.18±0.04	0.19±0.04	0.17±0.02	0.21±0.03	0.17±0.03
2-Methyl propionic acid	0.05±0.02	0.08±0.01	0.10±0.01	0.13±0.02	0.14±0.03	0.08±0.00	0.08±0.01	0.17±0.02	0.07±0.01	0.10±0.03	0.05±0.01	0.76±0.05	0.18±0.03
Ethyl butyrate	1.02±0.08	1.34±0.07	1.31±0.11	1.28±0.15	0.52±0.07	0.45±0.06	0.57±0.08	1.10±0.07	0.99±0.09	1.08±0.07	0.53±0.07	1.22±0.12	1.23±0.10
Ethyl lactate	3.26±0.77	3.75±0.17	3.47±0.24	3.27±0.13	2.74±0.11	2.65±0.13	2.72±0.15	5.24±0.12	2.16±0.14	2.29±0.15	2.58±0.10	2.26±0.13	2.77±0.16
3-Methyl butyric acid	0.30±0.06	0.28±0.04	0.33±0.04	0.32±0.02	0.27±0.05	0.28±0.03	0.37±0.05	0.27±0.05	0.18±0.04	0.16±0.02	0.17±0.03	0.15±0.02	0.27±0.04
2-Methyl butyric acid	0.38±0.07	0.44±0.05±	0.46±0.05	0.46±0.07	0.41±0.03	0.47±0.02	0.45±0.03	0.34±0.04	0.33±0.05	0.27±0.03	0.36±0.03	0.27±0.04	0.40±0.05
n-Hexanol	0.52±0.06	0.55±0.06	0.55±0.03	0.55±0.08	0.53±0.05	0.54±0.04	0.52±0.05	0.56±0.05	0.47±0.08	0.50±0.06	0.55±0.07	0.80±0.07	0.51±0.06

Ethyl valerate	0.13±0.03	0.15±0.02	0.21±0.02	0.41±0.06	0.10±0.01	0.10±0.01	0.11±0.02	0.14±0.02	0.09±0.01	0.10±0.01	0.10±0.01	0.12±0.02	0.10±0.01
Ethyl caproate	2.28±0.15	2.45±0.06	2.07±0.08	1.73±0.10	1.35±0.07	1.06±0.08	1.06±0.08	2.15±0.14	1.36±0.02	1.61±0.14	1.20±0.10	1.19±0.08	0.98±0.07
Ethyl hexadecanoate	0.65±0.13	0.44±0.07	0.52±0.03	0.67±0.06	0.35±0.04	0.24±0.04	0.22±0.03	0.87±0.06	0.62±0.10	0.76±0.08	0.54±0.04	0.65±0.07	0.50±0.06
Ethyl linoleate	0.11±0.03	0.21±0.03	0.18±0.01	0.15±0.02	0.06±0.00	0.13±0.03	0.11±0.01	0.12±0.02	0.12±0.02	0.15±0.02	0.17±0.02	0.11±0.01	0.11±0.01
Ethyl oleate	0.13±0.04	--	0.04±0.01	0.15±0.02	0.07±0.01	--	--	0.17±0.03	0.15±0.02	0.19±0.02	0.18±0.04	0.14±0.03	0.13±0.02

Note: In the row of sample, K represent blank culture medium without inoculation; The numbers "0, 1, 5, 13, 20 and 27" represent day 0, day 1, day 5, day13, day20 and day27 of fermentation, respectively; "--" represent not detected.
