# Supplementary material for: Study on the Effect of Key Genes ME2 and adhE during Luzhou-Flavor Baijiu Brewing
Source: Foods. 2022 Feb 26;11(5):700. doi: 10.3390/foods11050700 (PMC8909148; doi:10.3390/foods11050700)
Supplement: Supplementary file 1 [file foods-11-00700-s001.zip › foods-1589793-supplementary/Table S3.pdf]

Table S3. Flavor substances contents produced by CtA6 and LbA7 in different liquid fermentation stages.

Flavor substances（mg/L）	K	CtA6-0	CtA6-1	CtA6-5	CtA6-13	CtA6-20	CtA6-27	LbA7-0	LbA7-1	LbA7-5	LbA7-13	LbA7-20	LbA7-27
L-Lactic acid	13.71±1.47	6.15±0.96	14.84±0.91	9.19±0.20	13.64±.092	12.53±0.61	12.41±0.80	14.34±0.56	12.78±0.90	14.35±1.16	13.65±0.86	11.86±0.78	24.09±1.47
Acetic acid	1358.34±109.50	1227.95±73.15	911.65±79.12	908.06±33.27	1237.76±74.86	1313.71±52.81	1198.85±98.12	1322.60±101.88	1460.17±133.35	1191.19±86.86	986.39±39.02	1165.39±96.70	2216.61±117.11
Butyric acid	6.11±0.60	1.21±0.13	5.55±0.13	0.23±0.05	7.96±0.24	2.22±0.13	0.62±0.09	19.78±0.86	2.90±0.16	12.19±0.96	4.05±0.22	2.60±0.25	7.68±0.44
Ethyl acetate	4.73±0.22	4.70±0.28	4.33±0.16	4.18±0.07	3.57±0.38	3.36±0.19	3.49±0.28	4.08±0.26	2.78±0.17	3.83±0.23	3.25±0.10	2.93±0.08	2.60±0.12
3-Methyl butanal	0.12±0.02	0.25±0.04	0.12±0.02	0.12±0.04	0.21±0.03	0.24±0.04	0.09±0.02	0.11±0.02	0.07±0.01	0.08±0.12	0.09±0.17	0.07±0.00	0.26±0.05
n-Butanol	1.05±0.16	0.95±0.15	0.84±0.05	0.86±0.07	0.78±0.09	0.75±0.06	0.84±0.09	0.87±0.06	0.55±0.05	0.79±0.08	0.72±0.03	0.57±0.03	0.65±0.07

3-Methyl butanol	0.22±0.02	0.21±0.02	0.17±0.02	0.13±0.02	0.18±0.04	0.14±0.02	0.12±0.02	0.18±0.04	0.11±0.01	0.17±0.02	0.26±0.04	0.19±0.03	0.11±0.02
Ethyl butyrate	0.54±0.05	0.56±0.09	1.50±0.11	0.85±0.06	0.46±0.08	0.39±0.03	0.51±0.07	1.46±0.14	0.30±0.02	1.52±0.10	1.49±0.09	0.35±0.04	0.29±0.05
Ethyl lactate	2.84±0.37	2.34±0.19	3.11±0.17	2.61±0.09	2.04±0.19	1.68±0.11	1.66±0.14	2.87±0.19	1.75±0.04	2.79±0.20	2.17±0.14	1.48±0.03	1.54±0.07
n-Hexanol	0.65±0.06	0.47±0.04	0.47±0.03	0.50±0.10	0.34±0.07	0.40±0.04	0.53±0.08	0.53±0.06	0.29±0.04	0.46±0.04	0.43±0.06	0.34±0.04	0.28±0.04
Ethyl valerate	0.12±0.02	0.16±0.03	0.12±0.02	0.13±0.03	0.12±0.02	0.11±0.03	0.08±0.01	0.16±0.03	0.11±0.02	0.10±0.01	0.09±0.01	0.08±0.01	0.08±0.02
Ethyl caproate	3.14±0.17	2.85±0.16	1.88±0.06	1.83±0.05	1.97±0.08	1.46±0.14	1.23±0.07	2.75±0.12	1.54±0.07	1.64±0.04	1.56±0.03	1.30±0.06	0.64±0.26
Ethyl hexadecanoate	0.45±0.07	0.74±0.07	0.66±0.06	0.48±0.05	0.83±0.10	0.77±0.05	0.84±0.07	0.85±0.06	0.56±0.05	0.46±0.09	0.71±0.06	0.24±0.03	0.13±0.02
Ethyl linoleate	0.26±0.05	0.15±0.02	0.14±0.02	0.11±0.03	0.18±0.04	0.16±0.02	0.15±0.02	0.21±0.02	0.21±0.03	0.20±0.04	0.16±0.03	0.06±0.01	--

Ethyl oleate	--	0.16±0.03	0.20±0.02	0.12±0.01	0.22±0.02	0.23±0.04	0.16±0.03	0.20±0.05	--	--	0.18±0.01	0.05±0.02	--

Note: In the row of sample, K represent blank culture medium without inoculation; The numbers "0, 1, 5, 13, 20 and 27" represent day 0, day 1, day 5, day13, day20 and day27 of fermentation, respectively; "--" represent not detected.
